# Effect of *p*Hyph, A novel antibiotic-free treatment, on bacterial vaginosis symptoms and vaginal microbiome composition

**DOI:** 10.1016/j.eurox.2026.100497

**Published:** 2026-10-08

**Authors:** Emilia Lahtinen, Luisa W. Hugerth, Gabriella Edfeldt, Annette Säfholm, Sten Kornfält, Helena Strevens, Ina Schuppe-Koistinen

**Affiliations:** a Centre for Translational Microbiome Research, Department of Microbiology, Tumor and Cell Biology (MTC), Karolinska Institutet, Stockholm, Sweden; b Gedea Biotech AB, Medicon Village, Lund, Sweden; c Department of Medical Biochemistry and Microbiology, Science for Life Laboratory, Uppsala University, Uppsala, Sweden; d Department of Obstetrics and Gynecology, Institute of Clinical Sciences, Skåne University Hospital, Lund University, Sweden

**Keywords:** Non-antibiotic therapy, Bacterial vaginosis, Dysbiosis, Lactobacilli, QPCR, Vaginal microbiome, Vaginal malodor

## Abstract

**Background:**

Bacterial vaginosis (BV) is the most prevalent vaginal infection among reproductive-age women and is associated with increased susceptibility to gynecological complications. Current antibiotic treatments achieve high initial cure rates but show high rates of recurrence, partly due to their inability to remove persistent biofilms. *p*Hyph is a novel, non-antibiotic treatment that lowers vaginal pH, promoting *Lactobacillus* growth and biofilm disruption.

**Objective:**

To evaluate the immediate and long-term effects of *p*Hyph on the vaginal microbiome, BV resolution, and symptom reduction.

**Study design:**

In a randomized, controlled, partially blinded study, 61 women with clinically diagnosed BV were randomized to immediate (IT, n = 35) or delayed (DT, n = 26) treatment. Vaginal swabs were collected before treatment, after 6 days, and during maintenance to Day 70. Microbiome composition was analyzed using a qPCR panel targeting 47 bacterial and 6 fungal species.

**Results:**

Compared to controls*, p*Hyph reduced the abundance of multiple BV-associated species and increased *Lactobacillus iners* following 6-day treatment (median 8.96 ×10^3^ [Q1–Q3: 3.10 ×10^3^–2.72 ×10^4^] vs. median 9.44 × 10² [Q1–Q3: 0.00–4.29 × 10 ³] normalized qPCR counts, p < 0.05). Additionally, *p*Hyph reduced BV-related symptoms, particularly fishy odor (p < 0.05). Treatment promoted a shift to CST III microbiome composition, and maintenance dosing was associated with sustained *Lactobacillus* abundance up to Day 70.

**Conclusions:**

*p*Hyph shows potential as an alternative BV treatment by reducing BV-associated pathogens while promoting lactobacilli. Extended dosing may enhance microbiome stability.

## Highlights

• *p*Hyph treatment reduced BV-associated bacteria compared to controls.
• BV-related symptoms, including fishy odor, were reduced following treatment.
• *Lactobacillus iners* abundance increased after 6-day treatment.
• Maintenance dosing was associated with sustained *Lactobacillus* abundance.

## 1. Introduction

Bacterial vaginosis (BV) is the most common cause of malodorous vaginal discharge, affecting 23–29% of reproductive age women, with an estimated annual economic burden of $4.8 billion [1], [2]. It is characterized by a dysbiotic vaginal microbiome (VMB), where *Lactobacillus*-dominated communities are replaced by diverse, anaerobe-rich communities. BV is associated with gynecological infections, pelvic inflammatory disease, HIV- and HPV-infections, and preterm birth [3], [4], [5], [6].

The VMB is commonly classified into community state types (CSTs) based on dominant species. CST I – III and V describe *Lactobacillus*-dominated VMB compositions, while CST IV and its subtypes describe a dysbiotic VMB [7]. Restoration of a *Lactobacillus* dominance is considered a key therapeutic goal in BV management.

Primary treatment strategies for BV rely on antibiotics metronidazole and clindamycin, which can achieve clinical cure in 80–90% of patients [8], [9]. However, recurrence rates can reach 50% within one year [10]. This may result from disruption of the microbial balance and incomplete elimination of BV-associated biofilms by antibiotics [11], [12], [13]. These limitations highlight the need for treatments that restore microbial balance and provide long-term cure.

Non-antibiotic interventions have been proposed to address these challenges. *p*Hyph is an intravaginal treatment containing glucono-delta-lactone and sodium-gluconate that gradually lowers vaginal pH [14]. This may support restoration of microbial balance by reducing BV-associated bacteria and promoting *Lactobacillus* growth.

Our previous studies suggested that *p*Hyph may improve BV symptoms and modulate the VMB [14], [15]. However, because of a biologically active comparator (isomalt) and lack of a control group, the study had to be repeated to evaluate *p*Hyph’s effect in a controlled setting [14]. Here, we performed an exploratory analysis of clinical and microbiome data from a randomized, partially blinded clinical trial that included an untreated control group instead of placebo. An expanded qPCR panel was used to better characterize the effects of *p*Hyph on the VMB.

## 2. Materials and methods

### 2.1 Ethical statement

This clinical trial was conducted in Sweden and approved by the Swedish Ethics Committee (Dnr 2023–05329–01, October 3rd, 2023), in accordance with institutional and regulatory requirements. Written informed consent was obtained from all participants prior to trial-related activities.

### 2.2 Study design

This was an exploratory analysis of a randomized, parallel-group, partially blinded clinical trial conducted in Sweden to evaluate the safety and efficacy of *p*Hyph for BV (NEFERTITI-2; ClinicalTrials.gov ID: NCT06123299). In contrast to previous studies, an untreated control group was included, as no inert comparator with a comparable dissolution profile to *p*Hyph was available. Clinicians performing gynecological assessments were blinded.

### 2.3 Recruitment and eligibility

Adult, post-menarchal, pre-menopausal, non-pregnant women aged ≥ 18 years seeking help for BV-symptoms were recruited. Participants agreed to avoid using intravaginal products and use condoms or hormonal contraception during the study period. Those who had received antibiotic treatment 14 days prior to enrollment were excluded.

### 2.4 BV-diagnosis

BV was diagnosed at enrollment (Day 0) using Amsel’s criteria, requiring ≥ 3 of the following criteria: (1) thin, homogeneous discharge, (2) clue cells (>20%), (3) vaginal pH > 4.5, and (4) positive whiff test following addition of 10% KOH. BV resolution (primary endpoint) was defined as absence of criteria 1, 2, and 4.

### 2.5 Randomization and study arms

Participants were randomized in 1:1 ratio to either immediate treatment (IT) or delayed treatment (DT) groups.

IT group: Patients self-administered *p*Hyph once daily for 6 days and were assessed for BV resolution on Day 7, followed by twice-weekly maintenance treatment until Day 25, when BV-resolution was reassessed. Resolved patients continued twice-weekly treatment until Day 70 or recurrence.

DT group: Patients received no treatment during the first six days and were assessed for BV-resolution on Day 7. Those not resolved initiated the same *p*Hyph treatment and maintenance regimen as the IT group.

Patients not achieving BV-resolution were offered rescue treatment (dequalinium chloride vaginal tablets) and exited the study. Further information on the sample size calculations, randomization, and interim analysis is available in Supplementary Methods.

### 2.6 Treatment

The mode of action of pHyph has been described previously [14]. Briefly, pHyph is an intra-vaginal tablet containing glucono-delta-lactone (GDL) and sodium gluconate (NaG), which lowers vaginal pH, thereby inhibiting BV-associated bacteria while promoting lactobacilli growth.

### 2.7 Sample collection

Vaginal swab samples were collected at Days 0 and 7 for the patients in the IT group (Omnigene Vaginal OMR-130, DNA Genotek Inc, Canada). Further vaginal swabs were collected during maintenance treatment on days 25, 55, and 70. In the DT, vaginal swab samples were collected on days 0, 7, 14 and during maintenance treatment on days 32, 62, and 77. Day 55/62 samples were self-collected and mailed to the clinical site. All samples were stored in –80°C directly after sampling or reception. Symptom data, treatment usability, and suspected recurrence were collected via a mobile app.

### 2.8 DNA extraction and vaginal microbiome profiling

DNA extraction was performed at Karolinska Institutet as previously described [14], [16]. Samples were analyzed using the PMP™ Vaginal Microbiome Health Panel (Bio-Me, Oslo, Norway), comprising TaqMan™ qPCR assays targeting 47 bacterial and 6 fungal species, selected to provide comprehensive insight into the VMB. Total bacterial load was estimated using 16S rRNA gene copy numbers. Samples were normalized to their total dsDNA concentration.

### 2.9 Statistical analysis

Statistical analyses were performed using R (v4.4.1), except for symptom and adverse events analyses, which were conducted by the clinical contract research organization. Differences between independent and paired samples were assessed using Wilcoxon rank-sum and signed-rank tests, respectively. A significance level of α = 0.05 was used, and p-values were adjusted using the Benjamini–Hochberg method.

Three samples with unreliable 16S quantification were excluded from all statistical comparisons based on qPCR data due to low yield, defined as lower counts than the total counts of all panel taxa per sample. In sensitivity analysis, these samples were included in the same comparisons.

CST classification was performed with VALENCIA [17]. The tool classifies CST IV into the following subgroups: IV-A (high abundance of *Gardnerella vaginalis* and BVAB1), IV-B (moderate abundance of *G. vaginalis* and *Fannyhessea vaginae*), and IV-C (high abundance of various anaerobes). Where measured qPCR targets did not have a direct species-level match in VALENCIA, abundances were aggregated to the genus level. One sample was excluded from CST figures due to a lack of a reliable CST classification (Day 25).

For visualization, log10 values were computed as log10 (normalized counts + 1). Relative abundances were calculated by dividing normalized species counts by the total panel species count per sample. Non-iners *Lactobacillaceae* abundance was estimated as the summed abundance of all *Lactobacillus*, *Limosilactobacillus* and *Ligilactobacillus* species, except *Lactobacillus iners*.

## 3. Results

### 3.1 Cohort description

The study design is shown in Fig. 1. A total of 61 patients were enrolled, all of whom were included in the symptom analysis. Following a planned interim analysis showing a significant difference in BV resolution at Day 7 (primary endpoint) between the treatment and control groups, recruitment was stopped at 61 patients.

**Fig. 1:**
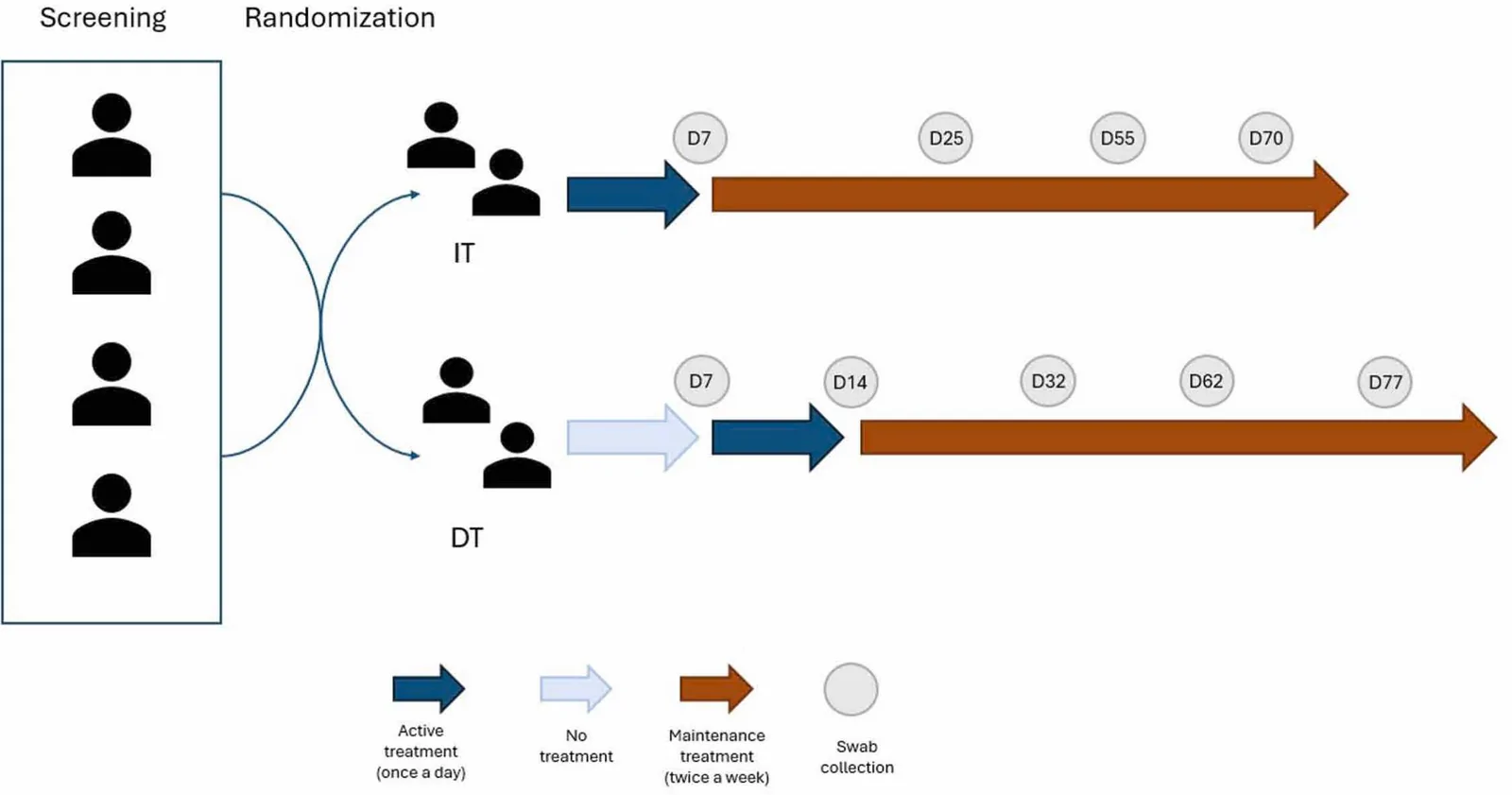
Schematic overview of the study design, including treatment allocation and timing of vaginal swab collection. Patients randomized to the immediate treatment group (IT) started *p*Hyph treatment immediately, while patients randomized to the delayed treatment group (DT) served as controls during the initial treatment period (Days 0–7) before receiving the same treatment regimen.

To assess the immediate effect of *p*Hyph on the VMB, matched qPCR samples collected before and after treatment were analyzed (n = 58, IT: Days 0 and 7, n = 34; DT Days 7 and 14, n = 24). Samples from withdrawn patients or patients with only one timepoint were excluded (n = 3). Long-term effects were evaluated using samples from the maintenance phase of both groups (Days 25, 55, and 70 relative to treatment start), excluding patients who received rescue treatment, withdrew, or were lost to follow-up.

Patient flow is illustrated in Fig. 2. Baseline characteristics for microbiome analysis are presented in Table 1 and did not differ significantly between groups.

**Fig. 2:**
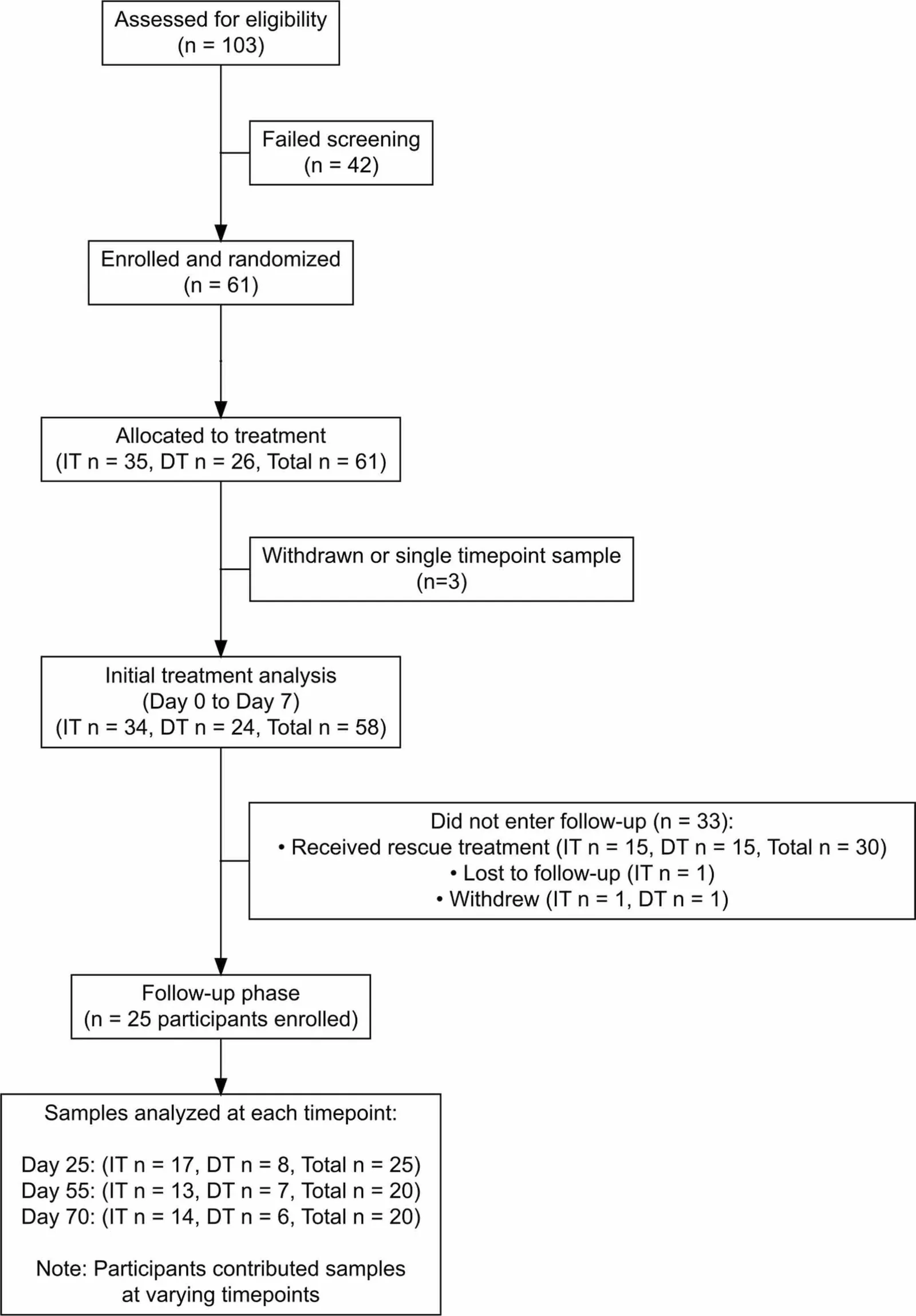
CONSORT flow diagram. This figure illustrates the number of participants screened, randomized, allocated to each study arm, completing follow‑up, and included in the final analyses.

**Table 1:** Baseline characteristics of enrolled patients.

Characteristic	DT N = 24^a^	IT N = 34^a^	p-value^b^
Clue cells			0.6
No	4 (17%)	9 (26%)	
Yes	20 (83%)	25 (74%)	
Discharge			> 0.9
No	1 (4.2%)	2 (5.9%)	
Yes	23 (96%)	32 (94%)	
Fishy odor smell			
Yes	24 (100%)	34 (100%)	
Age	27 (24, 44)	28 (24, 35)	0.6
Race			0.4
African descent	0 (0%)	1 (2.9%)	
Asian or Pacific Islander	0 (0%)	1 (2.9%)	
Middle Eastern	1 (4.2%)	0 (0%)	
Mixed/multiracial	1 (4.2%)	0 (0%)	
South American	0 (0%)	1 (2.9%)	
White/Caucasian	22 (92%)	31 (91%)	
Nugent Score			> 0.9
0–3	1 (4.2%)	2 (5.9%)	
4–6	1 (4.2%)	1 (2.9%)	
7–10	22 (92%)	31 (91%)	
Number of previous BV infections			0.9
> 3	8 (33%)	12 (35%)	
0–3	14 (58%)	18 (53%)	
missing	2 (8.3%)	4 (12%)	
pH	5.40 (5.15, 6.10)	5.33 (5.06, 5.60)	0.2

a n (%); Median (Q1, Q3)

b Pearson's Chi-squared test; Wilcoxon rank sum test

### 3.2 Microbiome shifts in treatment and control groups

We compared VMB changes between treatment (IT) and control group (DT) to confirm previously reported effects of *p*Hyph following 6-day treatment [14]. Before treatment, no significant differences in species abundances were observed (Supplementary Figure 1). By Day 7, the IT group showed significantly lower abundances of *G. vaginalis*, *Sneathia vaginalis*, *Amygdalobacter nucleatus*, *Dialister micraerophilus*, *Mobiluncus curtisii*, *Hoylesella timonensis*, *Porphyromonas uenonis*, and *Porphyromonas asaccharolytica* compared to the control group (all p < 0.05; Fig. 3). In contrast, *L. iners* abundance significantly increased in the IT group (p < 0.05; Fig. 3).

**Fig. 3:**
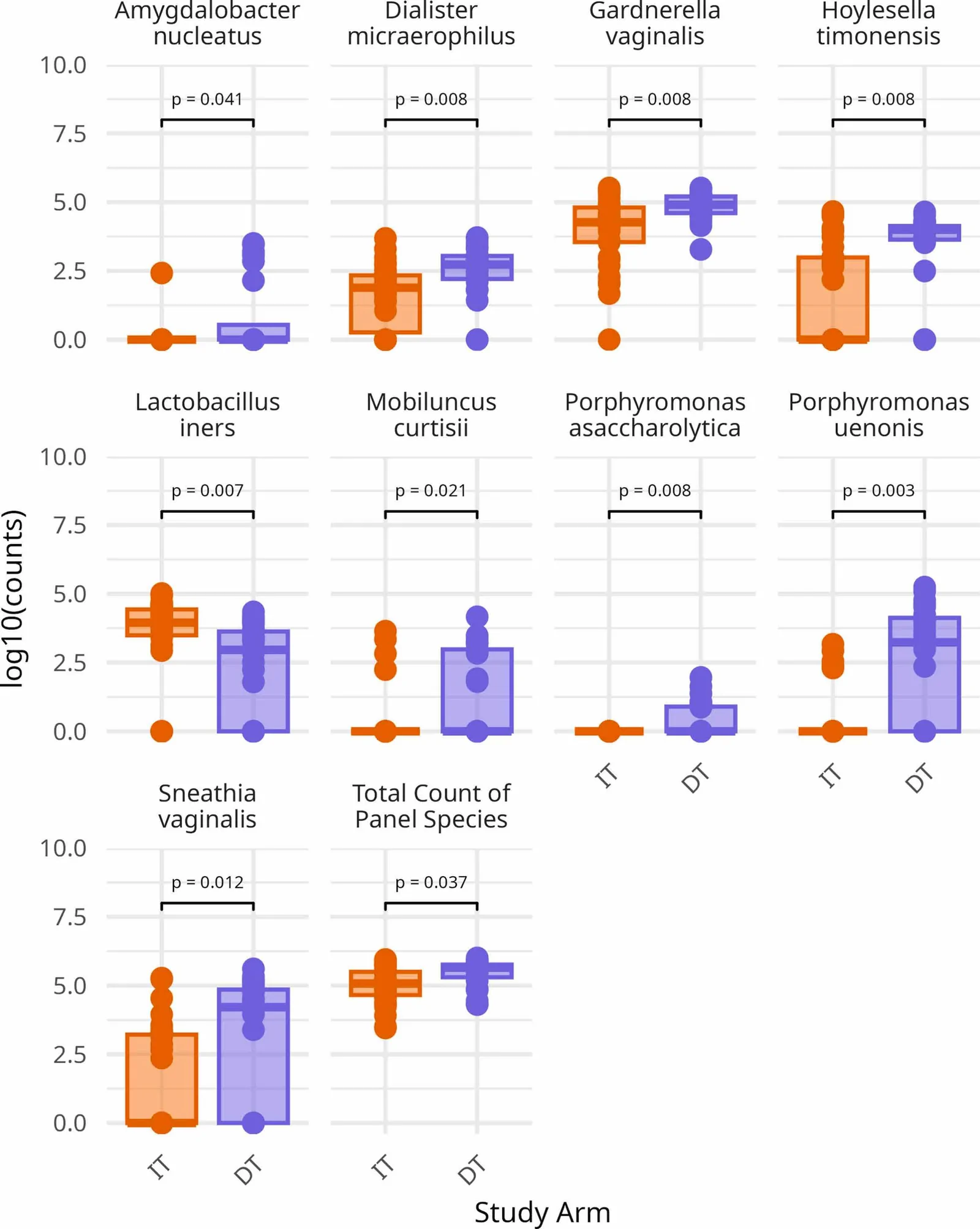
Species with significantly different abundance between treated (IT) and control (DT) groups at Day 7. Values are displayed on a log10(count + 1) scale. Statistical significance was assessed using the Wilcoxon rank-sum test with Benjamini–Hochberg-adjusted p-values (p < 0.05).

### 3.3 pHyph's impact on BV symptoms

Compared to DT group, *p*Hyph treatment significantly reduced fishy odor after six days (p < 0.05; Table 2). IT patients also showed fewer signs associated with the Amsel criteria; increased absence of clue cells (p < 0.05; Table 2) and more negative whiff tests (p < 0.05; Table 2). Vaginal pH also decreased significantly more in the IT group compared to the DT group (p < 0.05, ANCOVA; Table 2).

**Table 2:** Comparison of BV symptom measures (self-reported and clinician-assessed), vaginal pH changes, and Nugent scores across treatment arms on Day 7.

Characteristic at Day 7	Treatment Group	Control Group	p-value*
*Patient reported symptoms*			
Absence of fishy odor	22/35 (65%)	2/23 (8.7%)	< 0.0001
*Amsel criteria*			
Absence of Discharge	12/34 (35%)	4/25 (16%)	0.1408
Absence of Clue cells	21/34 (62%)	7/25 (28%)	0.0170
Negative whiff test	12/34 (35%)	1/25 (4%)	0.0043
pH ≤ 4.5	7/34 (21%)	2/25 (8%)	0.2776
*pH change from Day 0*			ANCOVA 0.0045
Mean (SD)	-0.44 (0.68)	-0.02 (0.74)	
Median (Min, Max)	-0.40 (-2.0, 0.8)	0.00 (-1.5, 2.1)	
*Nugent score*			
≤ 3	11/34 (32%)	1/25 (4%)	0.0087
< 7	18/34 (53%)	4/25 (16%)	0.0059

* If not otherwise specified, Fisher's exact test. Amsel criteria were assessed by the study clinician; self-reported symptoms were recorded using patient questionnaires.

On Day 7, BV resolution based on Amsel criteria was 21% in IT group (Supplementary Table 1, Panel A). Using Nugent score cutoffs 3 and 7, resolved BV rates were 32% and 53%, respectively (Supplementary Table 1, Panel A). By Day 25, BV resolution based on Amsel criteria increased to 45% across the cohort (Supplementary Table 1, Panel B). No patients were cured in the control group without treatment.

### 3.4 Impact of pHyph on the vaginal microbiome across the cohort

To further characterize *p*Hyph-associated microbiome changes, we combined before- and after-treatment samples across both groups based on treatment timing (IT: Days 0 and 7; DT: Days 7 and 14).

*p*Hyph treatment was associated with significant decreases in the abundance of multiple species (Fig. 4A), including *Amygdalobacter indicium* (BVAB2)*, Ca. Lachnocurva vaginae* (BVAB1), *F. vaginae*, and *Prevotella disiens* (all p < 0.05; Fig. 4B). The overall bacterial load (16S) also decreased (p < 0.05; Fig. 4B). Sensitivity analysis including samples with low 16S quantification produced comparable results. Conversely, abundance of *Candida albicans* increased (p < 0.05; Fig. 4B).

**Fig. 4:**
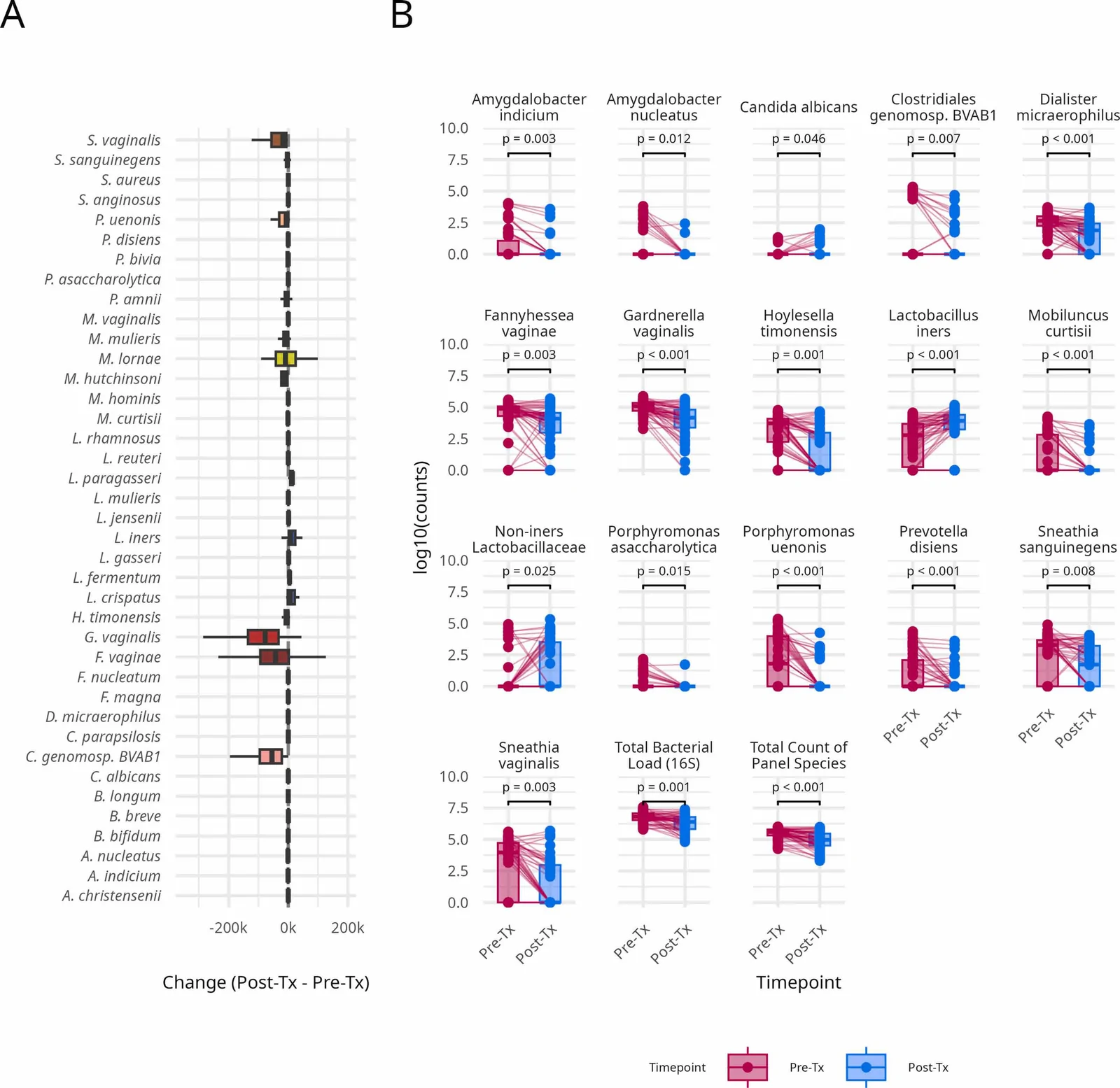
Changes in species abundance following *p*Hyph treatment based on paired samples before and after treatment. (A) Change in abundance of individual species included in the panel. (B) Change in abundance for species with statistically significant differences following treatment. Values in (A) represent changes in normalized absolute counts, while values in (B) are displayed on a log10(count + 1) scale. Statistical significance was assessed using the Wilcoxon signed-rank test with Benjamini–Hochberg-adjusted p-values (p < 0.05). Paired samples are connected by lines. Pre-Tx, before treatment; Post-Tx, after treatment.

*L. iners* and total non-iners *Lactobacillaceae* abundances increased following treatment (p < 0.05; Fig. 4B). Number of patients with detectable *Lactobacillaceae* also increased, although the change was not significant (Supplementary Figure 2 A). The increase in *Lactobacillaceae*-abundance was mainly observed in patients with detectable counts at baseline (Supplementary Figure 2B).

### 3.5 Vaginal microbiome stability after treatment

CST classification was used to assess overall microbiome dynamics. Before treatment, 93.1% (54/58) of patients belonged to CST-IV subgroups (Fig. 5A). Following 6-day treatment, CST IV-B remained the most common CST (30/58, 51.7%). Among those who shifted, over half transitioned to CST III (17/26, 65.4%) and in total 23 patients (23/26, 88.5%) moved to a *Lactobacillus*-dominated CST. CST III was the most common *Lactobacillus*-dominated CST, and its prevalence remained stable during the maintenance treatment (Fig. 5A).

**Fig. 5:**
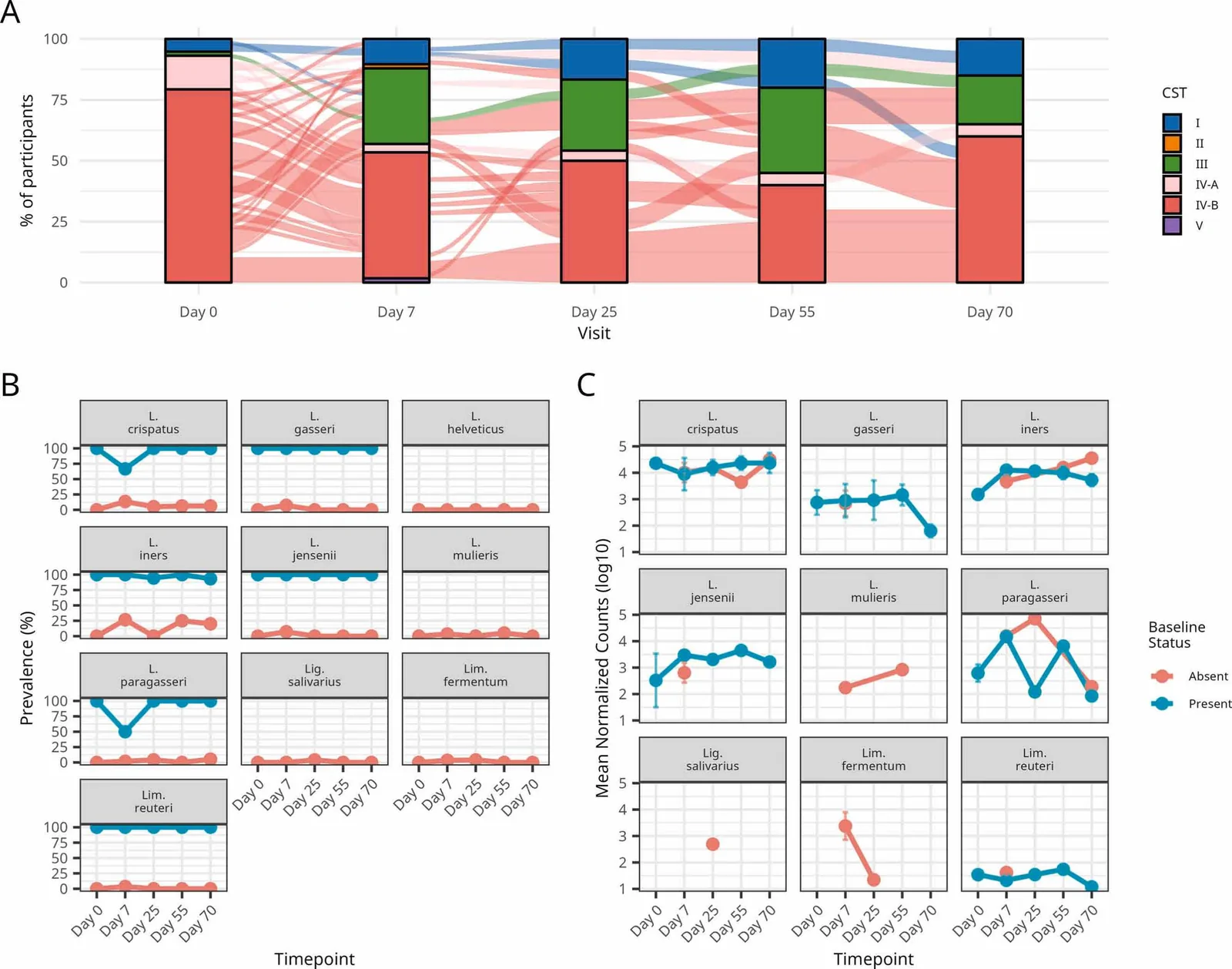
Changes in *Lactobacillaceae* abundance during the maintenance period. (A) CST transitions between timepoints. (B) Percentage of patients with detectable *Lactobacillaceae* over time, stratified by baseline colonization status before treatment (present or absent). (C) Changes in species abundance over time in colonized patients, stratified by baseline status. Each point represents the mean of normalized counts for patients with detectable counts at each timepoint.

To further examine microbiome stability, *Lactobacillus* species and other *Lactobacillaceae* abundances were assessed. Many species remained detectable during the maintenance treatment in baseline-colonized patients while baseline-negative patients showed minimal acquisition (Fig. 5B). Abundances among colonized patients remained generally stable (Fig. 5C). At the species level, responses varied. Specifically, *L. iners* maintained both prevalence and stable abundance, consistent with CST III dominance (Supplementary Figure 3). Interestingly, *L. crispatus* increased in a subpopulation of patients after immediate treatment and continued to increase during maintenance treatment.

### 3.6 Safety

*p*Hyph was well tolerated with no reported Serious Adverse Events (SAEs). Early adverse events were mainly related to vulvovaginal symptoms, such as burning, discomfort, and pruritus (Supplementary Table 2, Panel A). During the maintenance treatment, minor AEs were reported, often unrelated to vulvovaginal symptoms (Supplementary Table 2, Panel B). No cases of VVC were observed.

## 4. Discussion

In this exploratory analysis of a randomized, parallel-group, partially blinded clinical trial, *p*Hyph treatment was associated with clinical and microbiological changes consistent with BV-resolution. Compared to controls, *p*Hyph significantly reduced multiple BV-associated species and increased *L. iners* abundance following the 6-day treatment. These microbiome shifts were accompanied by reductions in BV-related symptoms, including fishy odor and clue cells, and a greater decrease in vaginal pH. Consistent microbiome changes were observed in within-patient analyses after all patients had received treatment, supporting the robustness of the findings.

Our results support previous evidence that *p*Hyph reduces BV-associated species, including *G. vaginalis*, and *M. curtisii* [14]. Notably, *D. micraerophilus*, a species recently linked to fishy odor [18], [19], significantly decreased in abundance. These findings suggest that *p*Hyph may target a broad range of taxa involved in BV pathogenesis. In the analysis including all treated patients, total bacterial load was also reduced, suggesting that *p*Hyph may alter microbiome composition and reduce overall microbial burden associated with BV and inflammation [20].

Consistent with previous observations, *p*Hyph increased the abundance of *L. iners*, a species commonly observed following antibiotic therapy [21], [22], [23]. *L. iners* dominance is often associated with a transitional state (CST III) that may facilitate recovery towards a VMB dominated by other lactobacilli [24], [25]. Its stable abundance during the maintenance phasesuggests persistent colonization, although its role in long-term transition toward dominance by other *Lactobacillus*-species, such as *L. crispatus*, requires further research.

When all treated patients were analyzed together, non-*iners Lactobacillaceae* abundance also increased, suggesting broader effects of *p*Hyph on lactobacilli and lactic acid bacteria. *L. crispatus* is a highly protective *Lactobacillus* species in the vagina due to its numerous health benefits [26]. Many patients with increased *L. crispatus* abundance were not detectably colonized at baseline, and the overall responses were heterogeneous. We hypothesize that decreases in BV-associated species abundances, and the total microbial load may create favorable conditions for it to flourish. Whether *p*Hyph can promote previously reported growth of *L. gasseri* and *L. jensenii* warrants further study [14].

In the same analysis, *C. albicans* abundance also increased, consistent with previous findings [14]. However, no VVC cases were observed, suggesting that the increase was limited to the non-pathogenic yeast form of *C. albicans*. This may be explained by *p*Hyph providing an alternative carbohydrate source that promotes yeast growth without inducing pathogenic transformation [14].

BV resolution based on Amsel criteria was lower than previously reported [14]. While Amsel-based resolution was modest, Nugent-based resolution rates were higher. This discrepancy likely reflects differences in assessment, as Amsel criteria rely on evaluation of clinical signs and were applied by different clinicians across studies, whereas Nugent scoring is standardized and performed by the same laboratory. Importantly, many patients reported symptom relief despite not meeting formal resolution criteria, underscoring the need to integrate clinical and microbiological outcomes in diagnosis.

Metronidazole and clindamycin remain first-line treatments for BV and are available in oral and intravaginal formulations [27]. Although effective, recurrence is common, potentially due to persistence of BV-associated biofilms and incomplete restoration of the VMB. Oral administration may also cause systemic adverse effects, and antibiotic treatment can increase antimicrobial resistance in the vagina [9], [10], [28]. Direct comparisons between *p*Hyph and antibiotic treatments are needed to define their relative clinical benefits and limitations.

Future studies should assess whether longer initial treatment improves BV resolution and further reduces dysbiosis. Controlled studies are also needed to assess the durability of the induced microbiome changes and their role in preventing recurrence. Additionally, evaluating *p*Hyph’s effectiveness in diverse populations, including pregnant women, and in combination with antibiotics or probiotics represents an important avenue for future research.

A major strength of this study is the inclusion of an untreated control group, enabling a robust assessment of treatment effects on the VMB. The broad qPCR panel, combined with total bacterial load measurements and extensive metadata, provided detailed insight into VMB dynamics. Several limitations should also be acknowledged. The analysis was limited to species included in the panel, which does not capture the entire complexity or functional potential of the VMB. Additionally, the study was only partially blinded, which may have influenced treatment outcomes. During the maintenance phase, the absence of a control group and the reduced sample size limited our interpretations of long-term microbiome dynamics. This study shows that *p*Hyph reduces the abundance of BV-associated bacteria while promoting a shift toward *Lactobacillus*-rich microbiome composition. These microbiome changes were accompanied by improvements in BV symptoms. These findings provide controlled evidence supporting the therapeutic potential of *p*Hyph in the treatment of BV.

## CRediT authorship contribution statement

**Emilia Lahtinen:** Writing – original draft, Visualization, Formal analysis. **Luisa W. Hugerth:** Writing – review & editing, Supervision, Formal analysis. **Gabriella Edfeldt:** Writing – review & editing, Supervision, Formal analysis. **Annette Säfholm:** Writing – review & editing, Funding acquisition. **Sten Kornfält:** Writing – review & editing, Project administration. **Helena Strevens:** Writing – review & editing, Supervision, Conceptualization. **Ina Schuppe-Koistinen:** Writing – review & editing, Supervision, Funding acquisition, Conceptualization.

## Declaration of Competing Interest

The authors declare the following financial interests/personal relationships which may be considered as potential competing interests: Emilia Lahtinen reports financial support was provided by Gedea Biotech AB. Helena Strevens reports financial support was provided by Gedea Biotech AB. Annette Safholm reports financial support was provided by Gedea Biotech AB. Sten Kornfalt reports financial support was provided by Gedea Biotech AB. Helena Strevens has patent issued to Gedea Biotech AB. Annette Safholm has patent issued to Gedea Biotech AB. Given her role as an editor at EJOG, Helena Strevens had no involvement in the peer review of this article and had no access to information regarding its peer review. Full responsibility for the editorial process for this article was delegated to another journal editor. -HS If there are other authors, they declare that they have no known competing financial interests or personal relationships that could have appeared to influence the work reported in this paper.

## Footnotes

Appendix A Supplementary data associated with this article can be found in the online version at doi:10.1016/j.eurox.2026.100497.
