# Supplementary material for: Effect of pHyph, A novel antibiotic-free treatment, on bacterial vaginosis symptoms and vaginal microbiome composition
Source: Eur J Obstet Gynecol Reprod Biol X. 2026 Oct 8;32:100497. doi: 10.1016/j.eurox.2026.100497 (PMC13696822; doi:10.1016/j.eurox.2026.100497)
Supplement: Supplementary file 1 [file mmc5.pdf]

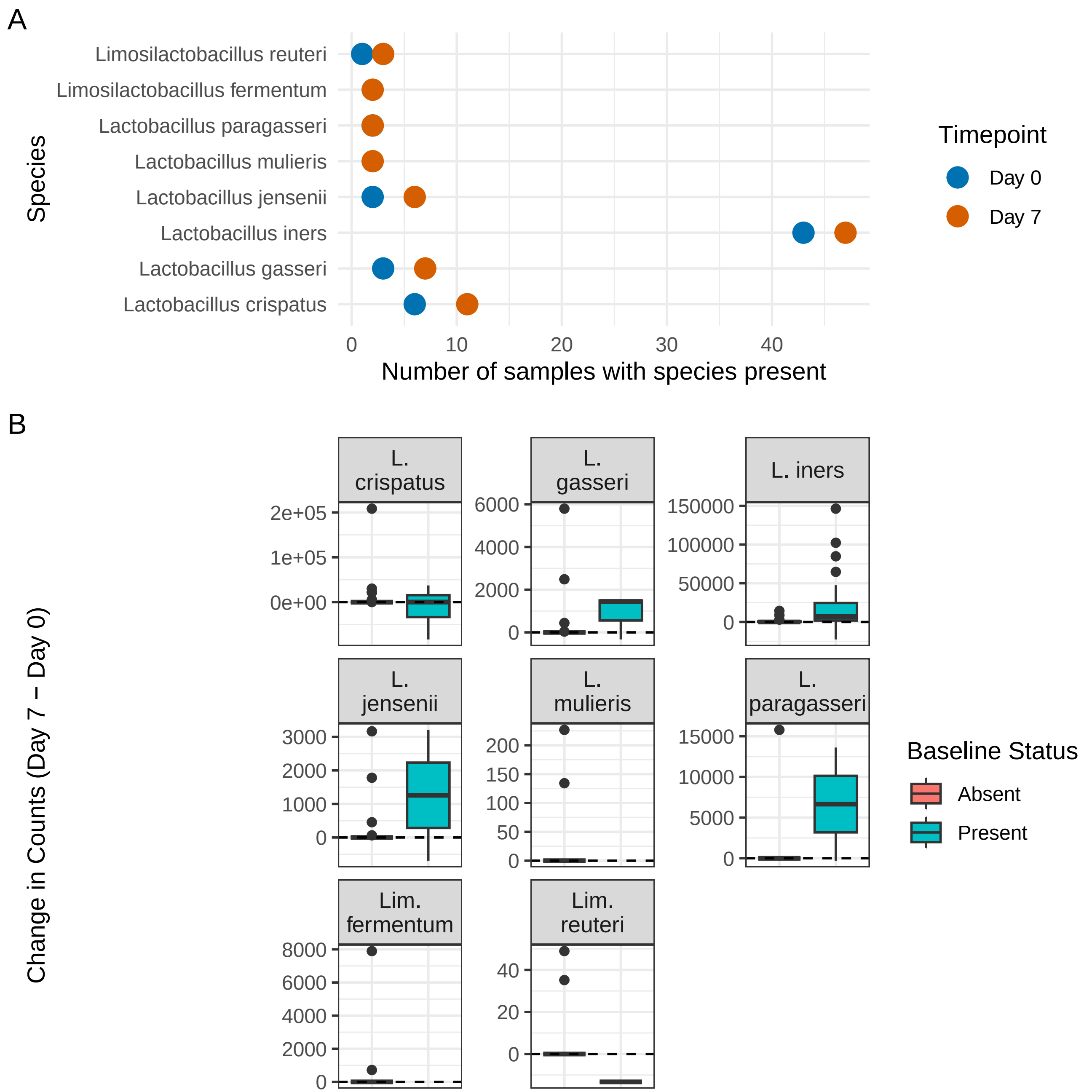
A
Limosilactobacillus reuteri
Limosilactobacillus fermentum
Lactobacillus paragasseri
Lactobacillus mulieris
Lactobacillus jensenii
Lactobacillus iners
Lactobacillus gasseri
Lactobacillus crispatus
Species
0
10
20
30
40
Number of samples with species present
Timepoint
Day 0
Day 7
B
L. crispatus
L. gasseri
L. iners
L. jensenii
L. mulieris
L. paragasseri
Lim. fermentum
Lim. reuteri
2e+05
1e+05
0e+00
6000
4000
2000
150000
100000
50000
3000
1000
200
150
100
50
15000
10000
5000
8000
40
20
0
Change in Counts (Day 7 – Day 0)
Baseline Status
Absent
Present
