# Supplementary material for: Effect of pHyph, A novel antibiotic-free treatment, on bacterial vaginosis symptoms and vaginal microbiome composition
Source: Eur J Obstet Gynecol Reprod Biol X. 2026 Oct 8;32:100497. doi: 10.1016/j.eurox.2026.100497 (PMC13696822; doi:10.1016/j.eurox.2026.100497)
Supplement: Supplementary file 2 [file mmc6.pdf]

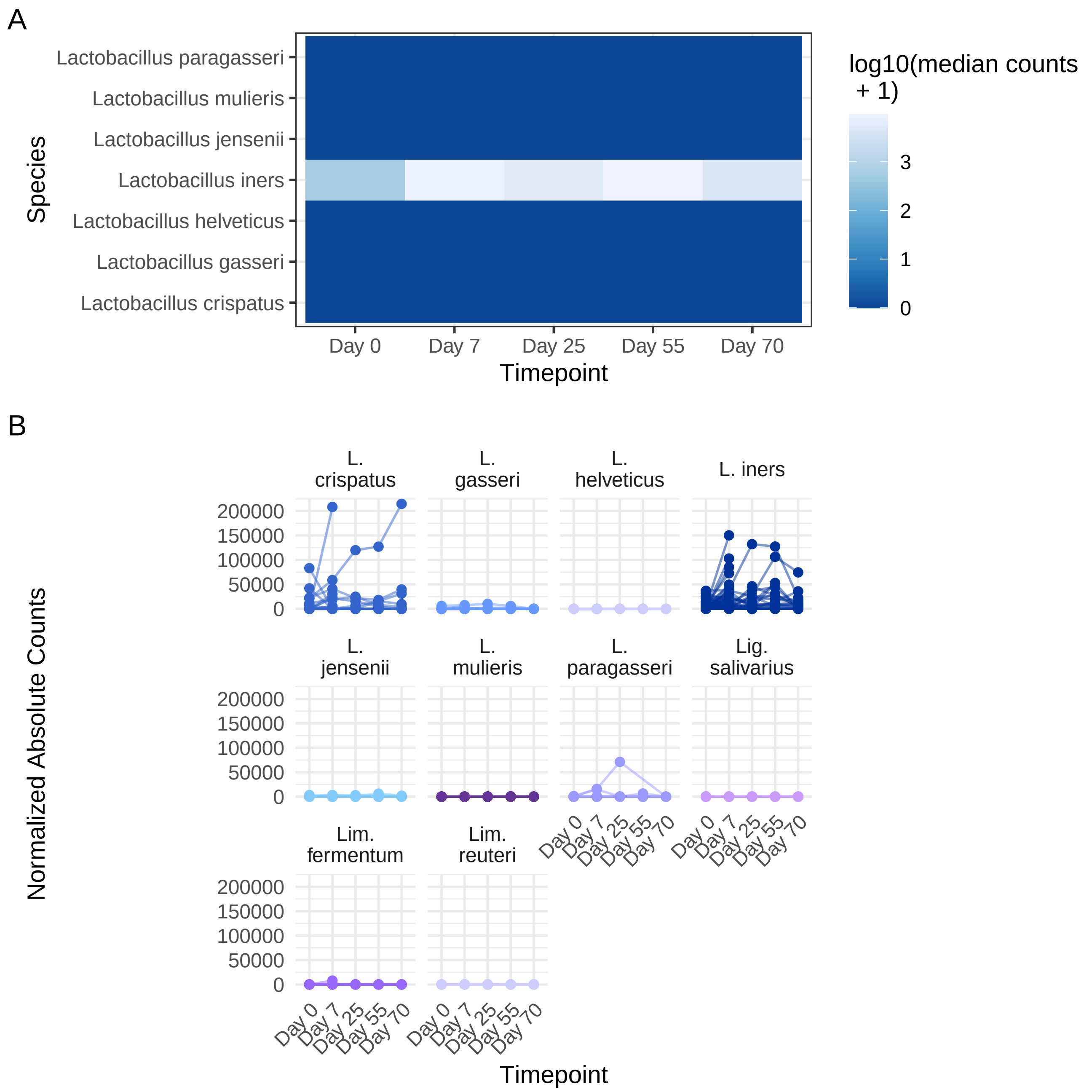
A
Lactobacillus paragasseri
Lactobacillus mulieris
Lactobacillus jensenii
Lactobacillus iners
Lactobacillus helveticus
Lactobacillus gasseri
Lactobacillus crispatus
Species
Day 0
Day 7
Day 25
Day 55
Day 70
Timepoint
log10(median counts + 1)
3
2
1
0
B
L. crispatus
L. gasseri
L. helveticus
L. iners
L. jensenii
L. mulieris
L. paragasseri
Lig. salivarius
Lim. fermentum
Lim. reuteri
200000
150000
100000
50000
0
Normalized Absolute Counts
Day 0
Day 7
Day 25
Day 55
Day 70
Timepoint
