# Supplementary material for: Effect of pHyph, A novel antibiotic-free treatment, on bacterial vaginosis symptoms and vaginal microbiome composition
Source: Eur J Obstet Gynecol Reprod Biol X. 2026 Oct 8;32:100497. doi: 10.1016/j.eurox.2026.100497 (PMC13696822; doi:10.1016/j.eurox.2026.100497)
Supplement: Supplementary file 3 [file mmc1.docx]

Supplementary Methods

**Sample Size**

The planned sample size was 82 to achieve a power of 80% at 5% significance level for detecting a difference in primary endpoint (BV-resolution based on Amsel criteria) between treated patients and the control group at Day 7. However, a pre-defined interim analysis was performed after 61 evaluable patients had enrolled in the study. The analysis achieved a statistical significance, and hence no further patients were enrolled.

**Randomization**

Randomization was performed in the EDC system, Viedoc™, using a static randomization scheme based on a computer-generated randomization list generated using SAS Proc Plan (SAS version 9.4). The randomization list was kept by the randomizer in a sealed envelope until the database lock. The randomization form in the eCRF was locked as soon as the randomization was performed. The randomization code was not broken until the time of interim analysis, after database lock, or in case of emergency unblinding.

**Interim Analysis**

The study included an interim analysis for the primary endpoint that was carried out after 61 patients had been enrolled, using the O’Brien - Fleming method with a significance level of 0.019. The final analysis used a significance level of 0.044. During the analysis, the primary endpoint was found to be significant between treated patients and the control group, and hence the study enrollment was discontinued at 61 patients.
