# Supplementary material for: Effect of pHyph, A novel antibiotic-free treatment, on bacterial vaginosis symptoms and vaginal microbiome composition
Source: Eur J Obstet Gynecol Reprod Biol X. 2026 Oct 8;32:100497. doi: 10.1016/j.eurox.2026.100497 (PMC13696822; doi:10.1016/j.eurox.2026.100497)
Supplement: Supplementary file 4 [file mmc2.docx]

**Supplementary Table 1. BV resolution at different time points**

**Panel A**

| **Resolution Method** | **IT Group** | **Control Group (DT)** | **p-value*** |
| --- | --- | --- | --- |
| *Amsel criteria* |  |  |  |
| Cure on Day 7 | 7/34 (21%) | 0/25 | 0.0172 |
|  |  |  |  |
| *Nugent score* |  |  |  |
| ≤3, Day 7 | 11/34 (32%) | 1/25 (4%) | 0.0087 |
| <7, Day 7 | 18/34 (53%) | 4/25 (16%) | 0.0059 |

*Fisher’s exact test.

**Panel B**

| **Resolution Method** | **IT Group** | **DT Group** | **All Treated** |
| --- | --- | --- | --- |
| *Amsel criteria* |  |  |  |
| Cure at 7 treatment days | 7/34 (21%) | 5/24 (21%) | 12/58 (21%) |
| Cure at 25 treatment days | 17/33 (52%) | 8/23 (35%) | 15/56 (45%) |
|  |  |  |  |
| *Nugent score* |  |  |  |
| ≤3, at 7 treatment days | 11/34 (32%) | 7/24 29%) | 18/58 (31%) |
| <7, at 7 treatment days | 18/34 (53%) | 11/24 (46%) | 29/58 (50%) |
| ≤3, at 25 treatment days | 8/32 (25%) | 0/22 | 8/54 (15%) |
| <7, at 25 treatment days | 13/32 (41%) | 5/22 (23%) | 18/54 (33%) |

Summary of BV cure rates stratified by treatment group. Panel A shows BV resolution on Day 7, when the DT group had not yet initiated treatment and served as the control. Panel B shows BV resolution at 7 and 25 days after treatment start (IT: Days 7 and 25; DT: Days 14 and 32). Amsel cure was defined as absence of homogenous discharge, clue cells, and positive whiff test. IT, immediate treatment; DT, delayed treatment.
