# Supplementary material for: Effect of pHyph, A novel antibiotic-free treatment, on bacterial vaginosis symptoms and vaginal microbiome composition
Source: Eur J Obstet Gynecol Reprod Biol X. 2026 Oct 8;32:100497. doi: 10.1016/j.eurox.2026.100497 (PMC13696822; doi:10.1016/j.eurox.2026.100497)
Supplement: Supplementary file 5 [file mmc3.docx]

**Supplementary Table 2. Most common adverse events during immediate and maintenance treatment periods.**

Panel A

|  | IT (N=35) |  | DT (N=26) |  | Total (N=61) |  |
| --- | --- | --- | --- | --- | --- | --- |
| System organ class  Preferred term | n (%) | m | n (%) | m | n (%) | m |
| Vulvovaginal burning sensation | 4 (11%) | 4 | 1 (3.8%) | 1 | 5 (8.2%) | 5 |
| Vulvovaginal discomfort | 2 (5.7%) | 2 | 2 (7.7%) | 2 | 4 (6.6%) | 4 |
| Vulvovaginal dryness | 1 (2.9%) | 1 | 1 (3.8%) | 1 | 2 (3.3%) | 2 |
| Vulvovaginal pruritus | 1 (2.9%) | 1 | 1 (3.8%) | 2 | 3 (5%) | 3 |
| Nasopharyngitis | 1 (2.9%) | 1 | 1 (3.8%) | 1 | 2 (3.3%) | 2 |

Panel B

|  | IT (N=35) |  | DT (N=26) |  | Total (N=61) |  |
| --- | --- | --- | --- | --- | --- | --- |
| System organ class  Preferred term | n (%) | m | n (%) | m | n (%) | m |
| Nasopharyngitis | 3 (8.6%) | 3 | 0 | 0 | 3 (4.9%) | 3 |
| Vulvovaginal pruritus | 0 | 0 | 1 (3.8%) | 2 | 1 (1.6%) | 2 |

Summary of adverse events (AEs) during the treatment period. Panel A shows AEs during the immediate treatment phase, and Panel B during the maintenance phase. No AEs were reported in the DT group up to Day 7. Most AEs occurred within 7–25 days after treatment start, with few events thereafter. n, number of patients; m, number of events. Percentages are based on the number of patients in each treatment group.
