# Supplementary material for: Effect of pHyph, A novel antibiotic-free treatment, on bacterial vaginosis symptoms and vaginal microbiome composition
Source: Eur J Obstet Gynecol Reprod Biol X. 2026 Oct 8;32:100497. doi: 10.1016/j.eurox.2026.100497 (PMC13696822; doi:10.1016/j.eurox.2026.100497)
Supplement: Supplementary file 6 [file mmc4.docx]

Supplementary Figure Captions

Supplementary Figure 1. Relative abundance of vaginal species per study arm at Day 0 and at Day 7. The relative abundance (%) was calculated by dividing the species counts by the sum of panel species for each sample.

Supplementary Figure 2. Change in *Lactobacillaceae* abundance following immediate treatment. A) Number of samples with detectable counts before and after treatment. For multiple species, the number of samples increases following treatment. B) The change in *Lactobacillaceae* abundance following treatment stratified by colonization at baseline. Box above zero indicates increase while below/around zero indicates no change. For the majority of the species, patients that were colonized by the species before the treatment began experienced a bigger increase after treatment compared to those who were not.

Supplementary Figure 3. Species and patient specific changes in the *Lactobacillaceae*-abundance over time. The species show very heterogenous responses to the treatment.
